# The complete chloroplast genome sequence of the medicinal plant *Mesona chinensis* (Labiatae)

**DOI:** 10.1080/23802359.2020.1788433

**Published:** 2020-07-09

**Authors:** Shiqiang Xu, Yufeng Li, Yan Zhao, Jihua Wang

**Affiliations:** a Guangdong Key Laboratory for Crops Genetic Improvement, Crops Research Institute, Guangdong Academy of Agricultural Sciences, Guangzhou, China; b Maize Research Institute, Luoyang Academy of Agriculture and Forestry Sciences, Luoyang, China

**Keywords:** *Mesona chinensis*, chloroplast genome, medicinal plant

## Abstract

*Mesona chinensis* is an important traditional Chinese medicine and edible plant resource in China. In this work, we sequenced the complete chloroplast genome of *M. chinensis* and researched its evolution. The genome size is 152,547 bp, with 37.89% GC content, including a large single copy region (LSC) of 83,482 bp, a small single copy region (SSC) of 17,725 bp and a pair of inverted repeats region (IRs) of 25,670 bp. The complete chloroplast genome was predicted to encode 131 genes, consist of 86 protein-coding genes, 37 tRNA genes and 8 rRNA genes. Phylogenetic analysis showed that *M. chinensis* was closely related to other Labiatae species *Ocimum tenuiflorum*.

Chloroplast (cp) is a semi-autonomous organelle in plant cells, which has relatively independent genetic material. Due to its simple structure and highly conserved, cp genome has been widely used in the research of molecular markers, genetic diversity and population variation (Daniell et al. 2016). In recent years, cp genomes of many medicinal plants have been reported, which will helpful to develop DNA barcode and improve the understanding of genetic structure (Qian et al. 2013; Zhang et al. 2016; Shen et al. 2017). However, the cp genome of *M. chinensis* is still unclear.

*Mesona chinensis*, belonging to the Labiatae family, is an importante conomic and medicinal plants, widely distroibuted in Fujian, Guangdong and Guangxi provinces, has the effects of antioxidation and hypolipidemic (Lin et al. 2017). In this study, the structural characteristics of cp genome of *M. chinensis* were described, which provides an important basis for further study. Firstly, fresh leaves of *M. chinensis* were collected from Guangdong Academy of Agricultural Sciences (Guangzhou, China). Then, the genomic DNA was extracted using plant genomic DNA kit (Omega), which was strored at Key Laboratory for Crops Genetic Improvement of Guangdong in Guangdong Academy of Agricultural Sciences (specimen code Lfc2019) and seuquenced on the Novaseq platform (Illumina, San Diego, CA). Finally, we assembled and annotated the cp genome by GetOrganelle and Geseq, respectively (Tillich et al. 2017; Jin et al. 2019).

The size of the cp genome was 152,547 bp, with 37.89% GC content, exhibited a typical quadripartite structure, consisted of a pair of IR regions (25,670 bp) separated by LSC region of 83,482 bp and SSC region of 17,725 bp (GenBank assession number MT328397.1). Total of 131 functional genes were predicted, including 86 protein-coding genes, 37 tRNA genes and 8 rRNA genes. The phylogenetic relationship of *M. chinensis* was constructed with the complete chloroplast genomes of other 26 species using maximum likelihood method by RaxML software (Stamatakis 2014). As shown in Figure 1, the position of *M. chinensis* was closely to *Ocimum tenuiflorum* with a bootstrap of 100%. The characterized cp genome sequence of *M. chinensis* will be facilitate future research on molecular breeding and genetic engineering.

**Figure 1.**
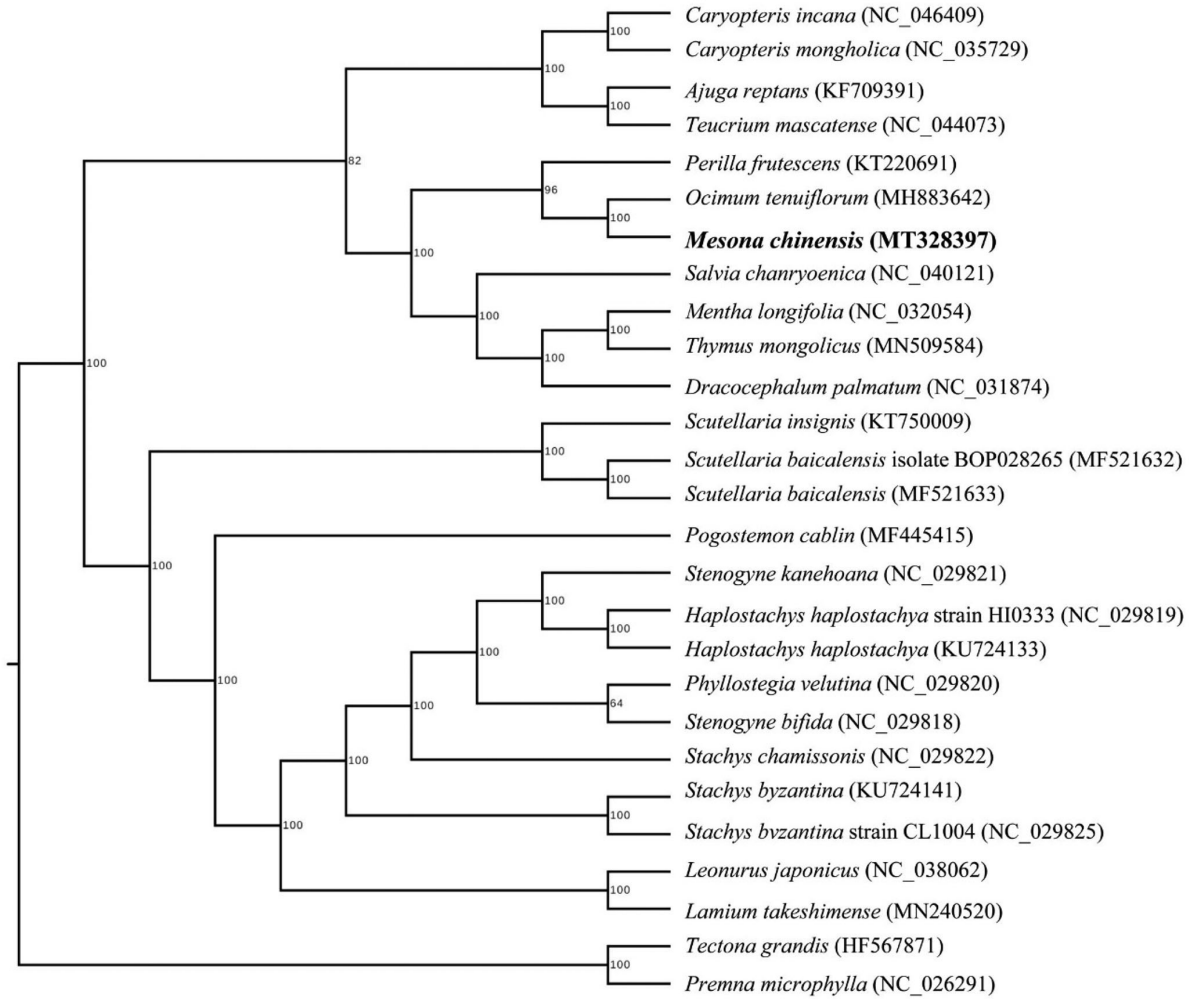
The phylogenetic tree of *M. chinensis* with other species based on the complete chloroplast sequence. Numbers above each node were bootstrap values. *Tectona grandis* and *premna microphylla* were set as outgroups.

## Data Availability

The data that support the findings of this study are openly available in [GenBank] at [https://www.ncbi.nlm.nih.gov/nuccore/MT328397.1], reference number [MT328397.1].
